# Nicotine Modulates Cognitive Function in *D*-Galactose-Induced Senescence in Mice

**DOI:** 10.3389/fnagi.2018.00194

**Published:** 2018-07-13

**Authors:** Alireza Majdi, Saeed Sadigh-Eteghad, Mahnaz Talebi, Fereshteh Farajdokht, Marjan Erfani, Javad Mahmoudi, Albert Gjedde

**Affiliations:** ^1^Neurosciences Research Center, Tabriz University of Medical Sciences, Tabriz, Iran; ^2^Departments of Clinical Research and Nuclear Medicine, Odense University Hospital, University of Southern Denmark, Odense, Denmark; ^3^Department of Neuroscience, University of Copenhagen, Copenhagen, Denmark; ^4^Department of Neurology and Neurosurgery, McGill University, Montreal, QC, Canada; ^5^Department of Radiology and Radiological Science, Johns Hopkins University, Baltimore, MD, United States

**Keywords:** aging, nicotine, learning and memory, oxidative stress, mitochondrial dysfunction, neurotrophic factors

## Abstract

Here, we tested the claim that nicotine attenuates the signs of brain dysfunction in the model of brain aging induced by *D*-galactose (DGal) in mice. We administered nicotine at doses of 0.1, 0.5 and 1 mg/kg by the subcutaneous (s.c.) or at 0.1 mg/kg by the intranasal (i.n.) routes in mice that had received DGal at the dose of 500 mg/kg subcutaneous (s.c.) for 6 weeks. We assessed animal withdrawal signs as the number of presented somatic signs, thermal hyperalgesia, elevated plus maze (EPM) and open field tests. We evaluated spatial memory and recognition with Barnes maze and novel object recognition (NOR) tests. We tested brain tissue for reactive oxygen species (ROS), mitochondrial membrane potential, caspase-3, Bax, Bcl-2, cytochrome C, brain-derived neurotrophic factor and nerve growth factor levels. Nicotine administration in model groups (0.5 mg/kg s.c. and 0.1 mg/kg i.n. doses) significantly attenuated impairment of spatial and episodic memories in comparison to normal saline-received model group. These doses also reduced mito-oxidative damage as well as apoptosis and raised neurotrophic factors level in model groups in comparison to normal saline-received model group. The 1 mg/kg s.c. dose nicotine revealed withdrawal signs compared with the other nicotine-received groups. Nicotine at specific doses and routes has the potential to attenuate age-related cognitive impairment, mito-oxidative damage, and apoptosis. The doses raise neurotrophic factors without producing withdrawal signs.

## Introduction

In aging, the progressive loss of physiological integrity and the decline of functional capacity lead to a range of disabilities. The cardinal aspects of aging of the brain include cognitive impairment, anxiety and depression. Oxidative stress, mitochondrial damage, apoptosis, neurotrophic factor loss, and cholinergic system dysfunction are known to be associated with aging of the brain and age-related deficits of learning and memory (Paradies et al., 2011; Gleichmann et al., 2012; Guarente, 2014; Ali et al., 2015; Richter et al., 2017). Multiple strategies have been tested with the goal of attenuating dysfunction of the aging brain, including the use of neuroprotective agents (He et al., 2009), inhibition of neuroinflammatory processes, prevention of oxidative stress (Zhang et al., 2009; Lu et al., 2010b; Ali et al., 2015) and activation of cholinergic neurotransmission (Lu et al., 2010b).

Nicotine is a nAChR agonist and pharmacological chaperone that stimulates cholinergic activity in the brain (Jackson et al., 2010; Sadigh-Eteghad et al., 2015a). It has been shown that nicotine improves working memory, executive function, and cognitive performance, both in human and animals (Rushforth et al., 2011; Jansari et al., 2013; Vafaee et al., 2015; Majdi et al., 2017). Nicotine also reduces reactive oxygen species (ROS) generation by brain mitochondria and prevents oxidative stress in a dose-dependent manner (Cormier et al., 2003; Guan et al., 2003). Nicotine further protects neurons against mitochondrial apoptosis (Garrido et al., 2001). There is evidence that nicotine is neuroprotective and regulates neurotrophic factors in the brain and that it affects the development and maturation of neurons (Xiaoyu, 2015). Experimental and clinical studies both, have revealed that nicotine administartion renders pro-cognitive effects (Bontempi et al., 2003; Myers et al., 2008). This finding has implications in the treatment of disorders which primarily affect cognition including Alzheimer’s disease (AD; Levin et al., 2006).

Nonetheless, it is clear that nicotine may induce dependency and subsequent withdrawal symptoms in animals and humans that can be prevented by use of limited doses and selective routes of delivery to the brain (Matta et al., 2007). Intranasal (i.n.) drug delivery is one such alternative to conventional routes of administration to the brain. The delivery is non-invasive, bypasses blood-brain barrier (BBB), allowing the drug to target the olfactory region as the direct avenue from nose to the brain. In the case of nicotine, the intranasal route is an alternative choice for delivery of nicotine to the brain (Farzampour et al., 2016; Pourmemar et al., 2017).

Chronic administration of *D*-galactose (DGal) to animals has consequences that mimic the characteristics of the aging brain and the related learning and memory impairment in humans (Kumar et al., 2010; Lu et al., 2010a; Lan et al., 2012; Tsai and Yin, 2012; Salehpour et al., 2017), with extensive application of this animal model to the study of the aging brain (Wei et al., 2005; Hsieh et al., 2009; Lu et al., 2010b; Zhou et al., 2013; Ali et al., 2015; Sadigh-Eteghad et al., 2017). It has been reported that DGal administration could recapulate the changes that happen during accelerated aging (Song et al., 1999). Also, evidence suggests that prolonged DGal-injected animals resemble their 16- to 24-month-old senescent animals (Gong and Xu, 1991; Li et al., 1995; Zhang et al., 1996). These characteristics make DGal-induced aging model a valid tool to recapitulate human aging in rodent (Song et al., 1999).

Here, we tested the claim that chronic nicotine administration attenuates memory impairment, apoptosis, and oxidative damage by cholinergic receptor activation, and that it may raise neurotrophic factors, independently of its addictive potential in a mouse model of aging of the brain induced by DGal.

## Materials and Methods

### Animals

Seventy-two male BALB/c mice weighing 25–30 g were obtained from Tabriz University of Medical Sciences laboratory animal care center. Animals were socially housed in standard polypropylene cages (five in each cage) under the controlled condition of constant humidity and temperature on a 12 h light/12 h dark schedule before and through study with access to water and standard pellet food *ad libitum*.

### Ethics Statement

All efforts were made to minimize animal suffering and the number of animals used. Also, all procedures were performed in accordance with the recommendations of the guide for the care and use of laboratory animals of the National Institutes of Health (NIH; Publication No. 85-23, revised 1985) and approved by the regional ethics committee of Tabriz University of Medical Sciences (IR.TBZMED.REC.1395.61).

### Experimental Procedures

**Figure 1:**
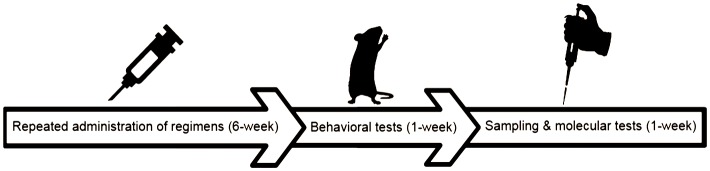
The experimental design and timescale of the study.

The animals were randomly divided into six groups with 12 mice in each; in the control group, mice did not receive any injection or treatment. In the DGal-induced aging group, for modeling of brain aging, mice were injected with DGal (500 mg/kg s.c. for 6 weeks; Sigma-Aldrich, St. Louis, MO, USA). Separate groups of DGal-injected mice received either NS or nicotine (Santa Cruz Biotechnology, Santa Cruz, CA, USA) through either s.c. (0.1, 0.5 and 1 mg/kg) or i.n. (0.1 mg/kg) routes for 6 weeks. For i.n. administration of nicotine drops containing 5–6 μl were administered through nasal mucosa with alternation between left and right nares for 2 min to reach the total desired volume (Pourmemar et al., 2017). At the end of the treatment administration period, the behavioral tests and biochemical analyses were performed (Figure 1). All tests were performed by an experimenter that was unaware of the identity of experimentations. All solutions were freshly prepared on the day of experimentation by dissolving drugs in NS (0.9% NaCl). All injections had a volume of 8 ml/kg body weight.

### Withdrawal Signs Assessment

Twenty-four hours after the administration of the last dose of treatments, the withdrawal signs of mice was assessed using the following tests (Damaj et al., 2003).

#### Somatic Signs

Mice were placed in the Plexiglas cages, and their behavior was recorded and controlled for somatic withdrawal signs including paw tremors, head shakes, writhing, retropulsion, scratching and Straub tail for 20 min.

#### Elevated Plus-Maze

To assess animals’ anxiety, elevated plus-maze (EPM) test was used. Briefly, EPM consisted of two open and two fenced arms that stretched from a central platform raised 60 cm from the ground. Mice were placed in the center of the maze, their behavior was recorded for 5 min and analyzed using EthoVision video tracking system, and the percentage of time spent in the open arms (%OAT) and entries to the open arms (%OAE) were extracted. After each animal removal, the apparatus was completely cleaned with a 70% ethanol to remove olfactory or intra-apparatus cues.

#### Open Field Test

Mice locomotor activity and anxiety were evaluated using open field test. The animals were placed in a black Plexiglas open-field box measuring 33 cm × 33 cm × 20 cm and their behavior was recorded using EthoVision video tracking system for 20 min. The total distance moved considered as a locomotor activity, and arena periphery locomotion was measured as anxiety index.

#### Thermal Hyperalgesia

The hotplate test was used to assess animals’ thermal hyperalgesia. The animal was placed in a cylindrical glass (width: 20 cm) on a hot plate apparatus, and the temperature maintained at 48°C. The reaction time for paw-licking or jumping was recorded as indices of thermal hyperalgesia.

### Learning and Memory Profile

#### Novel Object Recognition

Novel object recognition (NOR) test was used to assess mice recognition memory. It was performed according to the previously explained method (Pourmemar et al., 2017). Briefly, the test apparatus was a black Plexiglas open-field box measuring 33 cm × 33 cm × 20 cm. One day before the training session, mice were placed in the test room for 15 min for acclimation, and then each animal was transferred to the test box for a 10-min habituation session. On the training session, two identical objects (A and A’) were placed in the box and introduced to the mice. The objects used were common and different in shape and texture, however, were counterbalanced in complexity. Then, each animal was placed in the middle of the box and the total time spent to explore each object was recorded over 10 min. The mice then returned to their home cage. The next day after training session mice were reintroduced to the same task, but one of the familiar objects applied during the training session was replaced by a novel object (B) for a single retention session. The mice were considered to be exploring when the animal nose was toward the object (a distance of ≤2 cm), and there was rearing up against it. After each trial, the test box and objects were cleaned with a 70% ethanol to eliminate the presence of any olfactory cues. The cut-off time for exploration of two objects was 20 s over a period of 10 min. The recognition memory was measured by the time spent exploring of novel object. Data were acquired using a video camera that was fixed above the center of the task apparatus and analyzed using a video tracking program Etho Vision™ (Noldus, Netherlands).

#### Barnes Maze

The Barnes maze was used to assess spatial learning and memory in the animals (Sunyer et al., 2007). The maze consisted of a circular platform 100 cm in diameter raised 50 cm from the ground, with 20 holes (hole diameter: 5 cm) around the maze. An escape box (20 cm × 15 cm × 5 cm) was placed under the target hole. The test was carried out in a room with special spatial cues located on the walls and a buzzer (80 dB) as a negative stimulus.

The Barnes maze test included habituation, acquisition and probe sessions lasting 6 days. The first day of the test comprised habituation session. During this session, the animal was located in the center of the maze in a black cylindrical start chamber. After 10 s, the start chamber was lifted, the buzzer was switched on, and the animal was gently guided to the escape box, the buzzer was turned off, and the mice stayed there for 1 min.

The consequent days consisted of four acquisition trials per day, divided by a 3-min interval for 4 days. In the spatial acquisition session, the same condition repeated and animals were free to explore arena and find the escape box for 3 min. After entering the box, the buzzer was turned off, and the mice remained there for 1 min. The last day of the experiment had one probe trial session lasting 3 min. The Barnes maze arena without the escape box was used to assess mice reference memory.

After each session and trial, the entire maze and escape box were cleaned with 70% alcohol to remove olfactory cues. The time that took the mice to find the escape box (latency time) during the training sessions, time spent in the target quadrant, and correct to error time (time spent in the target hole/time spent in the error holes) during the probe session were evaluated using EthoVision™ software.

### Oxidative Damage to Mitochondria

#### Brain Tissue Sampling and Isolation of Mitochondria

Twenty-four hours after the last behavioral test, mice were decapitated after deep anesthesia with ketamine (100 mg/kg) and xylazine (10 mg/kg). The animal brain except cerebellum was then extracted and transferred to the freezing beaker for further evaluations and biochemical assays. For mitochondria isolation, fresh brain samples placed in ice-cold isolation buffer containing 200-mM mannitol, 70-mM sucrose, 10-mM HEPES, and 2-mM EDTA, pH 7.5. Thereafter, the tissue was homogenized in ice-cold extraction buffer having 2 mg/mL albumin (10% w/v). The samples were centrifuged at 600 *g* in 4°C for 5 min. Then, the supernatant was transferred into another tube and centrifuged at 12,000 *g* in 4°C for 15 min. Finally, the pellet was resuspended in storage buffer containing 10-mM HEPES, pH 7.4, 250-mM sucrose, 1-mM ATP, 0.08-mM ADP, 5-mM sodium succinate, 2-mM K_2_HPO_4_ and 1-mM DTT. Protein level was determined by Bradford method (Bradford, 1976).

#### Mitochondrial Membrane Potentials

Mitochondrial membrane potentials (MMP) or Δψm changes were detected using JC-1 vital dye (Mitochondria Staining Kit; Sigma-Aldrich, St. Louis, MO, USA). Under normal condition, the concentration of dye in the mitochondrial matrix produces red fluorescent. Any situation during which the MMP is dissipated results in shifting from red to green fluorescence responsible for J-aggregates and JC-1 monomers respectively. Briefly according to the kit instructions, JC-1 stain (0.6 μM) was used. Fluorescence intensity in the stained samples was measured by the fluorimetric assay. The ratio of red (*λ*_ex_ = 490 nm, *λ*_em_ = 590 nm) to green (*λ*_ex_ = 488 nm, *λ*_em_ = 530 nm) fluorescence intensity was considered as the Δψm which was normalized to the sample proteins.

#### Mitochondrial ROS Generation

To determine the mitochondrial ROS level, the fluorescent vital dye dichlorohydro-fluorescein diacetate (DCFDA) was used (Novalija et al., 2003). This fluorescent probe (2-μM at 37°C for 20 min) is oxidized by mitochondrial ROS to Dichlorodihydrofluorescein (DCF), and the resulting fluorescence intensity was measured according to the DCF spectral characteristics (*λ*_ex_ = 485 nm, *λ*_em_ = 530 nm). The resulting ROS level was expressed as fluorescence intensity which was normalized to the sample proteins.

#### Apoptosis Markers and Cytochrome *C*

Western blotting was performed using the previously explained method (Sadigh-Eteghad et al., 2015b). For total and cytosolic fractions preparations, 100 mg of fresh brain tissue was homogenized using ice-cold mitochondria isolation buffer [200 mM mannitol, 80 mM HEPES-KOH (pH 7.4), and the protease inhibitor cocktail]. Homogenates were centrifuged at 750× for 10 min at 4°C. After removing half of the supernatants that were used as total fractions, the rest of the supernatants were centrifuged at 12,000× *g* for 20 min at 4°C which was the mitochondria free fraction including cytosol.

Radioimmunoprecipitation assay (RIPA) buffer containing protease inhibitors plus total or cytosolic fractions were homogenized. The protein concentration of the sample was determined using the Bradford assay (Li et al., 2008). Electrophoresis was performed using 12.5% polyacrylamide gel, and the isolated proteins were transferred onto a polyvinylidene difluoride (PVDF; Roche, United Kingdom). These membranes were then incubated with the primary antibodies (Santa Cruz Biotechnology, Santa Cruz, CA, USA) including anti-caspase-3 (1:500, sc-7148), anti-Bcl-2 (1:500, sc-492), anti-Bax (1:500, sc-493), and anti-cytochrome C (1:500, sc-7159) antibodies. After three times of washing lasting 5 min each, the membranes were incubated with the horseradish peroxidase conjugated goat anti-rabbit IgG secondary antibody for 60 min (1:5000, sc-2004). The membranes were finally positioned in ECL prime western blotting detection reagent (Amersham, United Kingdom) and the resulting signals visualization was achieved using Kodak autoradiography film (Kodak, Rochester, NY, USA). Anti *β*-actin (1:500, sc-130656) antibody was applied for internal control of the procedure. The quantification of the signal intensity of each band was performed using ImageJ 1.62 software (National Institutes of Health, Bethesda, MD, USA) and was normalized to the matching internal control.

### Brain BDNF and NGF

ELISA method was used to determine mouse BDNF (Elabscience Biotechnology, China) and brain NGF (Shanghai Crystal day Biotech, China) levels in the brain homogenate. All procedures and calculations were performed using a commercial kit according to the manufacturer’s instructions. Briefly, the tissues were minced to small pieces and rinsed in ice-cold PBS (0.01 M, pH = 7.4) to eliminate excess blood entirely. Tissue pieces were weighed and then homogenized in PBS with a glass homogenizer on ice. To further break the cells, the suspension was sonicated with an ultrasonic cell disrupter. The homogenates were then centrifuged for 5 min at 5000× *g* to get the supernatant.

### Statistics

Descriptive data were expressed as mean ± standard error of mean (SEM). Comparison of different groups was carried out by a one-way analysis of variance (ANOVA) followed by the *post hoc* Tukey test or two-way ANOVA when applicable. All analyses were performed using GraphPad Prism software (version 7 for Windows; GraphPad Software Inc., La Jolla, CA, USA). For all comparisons, *p* < 0.05 was considered to be significant.

## Results

### Withdrawal Signs

#### Somatic Signs

**Figure 2:**
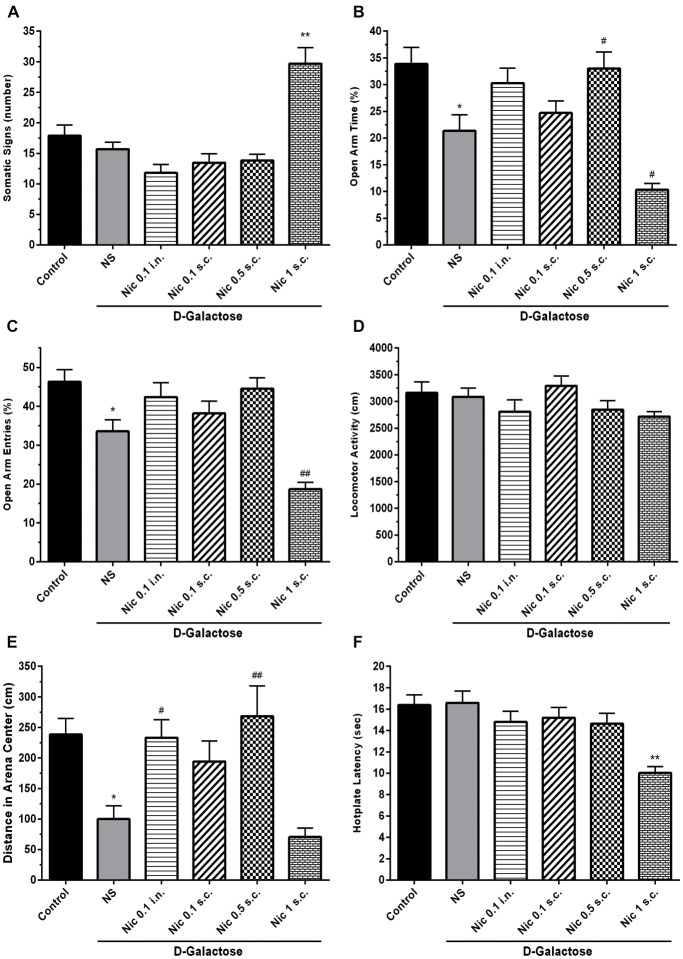
Withdrawal signs in study groups. **(A)** Number of somatic signs, **(B)** percentage of time spent in the open arms of EPM, **(C)** percentage of entries to the open arms of EPM, **(D)** locomotor activity, **(E)** distance moved in the center of the arena and **(F)** hotplate latency times. Each bar represents the mean ± standard error of mean (SEM), (*n* = 12). Significant differences tested by one-way analysis of variance (ANOVA) followed by Tukey’s *post hoc* test; *p < 0.05 and **p < 0.01 compared to the control group. ^#^*p* < 0.05 and ^##^*p* < 0.01 compared to the DG+NS group; NS, normal saline; DG, *D*-galactose; Nic, nicotine; s.c., subcutaneous; i.n., intranasal; EPM, elevated plus maze.

One-way ANOVA showed a statistically significant difference between groups in the somatic signs presented by the animals (*F*_(5,66)_ = 18.55, *p* < 0.001). *Post hoc* analysis revealed that spontaneous withdrawal of nicotine had not a significant impact on the number of somatic signs at the administered doses except for 1 mg/kg s.c. dose which significantly increased the number of somatic signs compared to other groups (*p* < 0.01; Figure 2A).

#### Elevated Plus Maze

We found statistically significant differences between groups in OAT (*F*_(5,66)_ = 11.70, *p* < 0.001) and OAE (*F*_(5,66)_ = 14.05, *p* < 0.001) tasks in elevated plus maze (EPM) test. Accordingly, *post hoc* analysis showed that chronic DGal+NS administration reduced both OAT and OAE in comparison to the control group (*p* < 0.05). On the other hand, chronic nicotine injection increased OAT at 0.5 mg/kg s.c. dose in DGal-received animals. Also, chronic nicotine administration at 1 mg/kg s.c. dose significantly decreased OAT and OAE compared with the DGal+NS group (*p* < 0.05 and *p* < 0.01 respectively). Other used doses of nicotine did not significantly affect OAT and OAE (*p* > 0.05; Figures 2B,C).

#### Open Filed Test

There was no significant difference between groups in the total distance moved in open filed test (*F*_(5,66)_ = 1.824, *p* = 0.12; Figure 2D). However, difference for the time spent in the center was significant between groups (*F*_(5,66)_ = 8.206, *p* < 0.001). Chronic DGal+NS treatment significantly reduced distance moved in the center compared to the control group (*p* < 0.05). However, chronic nicotine administration at 0.5 mg/kg s.c. and 0.1 mg/kg i.n. doses remarkably increased distance moved in the center in comparison to the NS-received DGal group (*p* < 0.01 and *p* < 0.05 respectively; Figure 2E).

#### Hotplate

We found a significant difference between study groups in hotplate latency time (*F*_(5,66)_ = 7.766, *p* < 0.001). Nicotine administration at 1 mg/kg s.c. dose significantly decreased latency time in hotplate test in comparison to other groups (*p* < 0.01). Other used doses of nicotine did not have an impact on the hotplate latency time (*p* > 0.05; Figure 2F).

### Learning and Memory Profile

#### Barnes Maze Test

**Figure 3:**
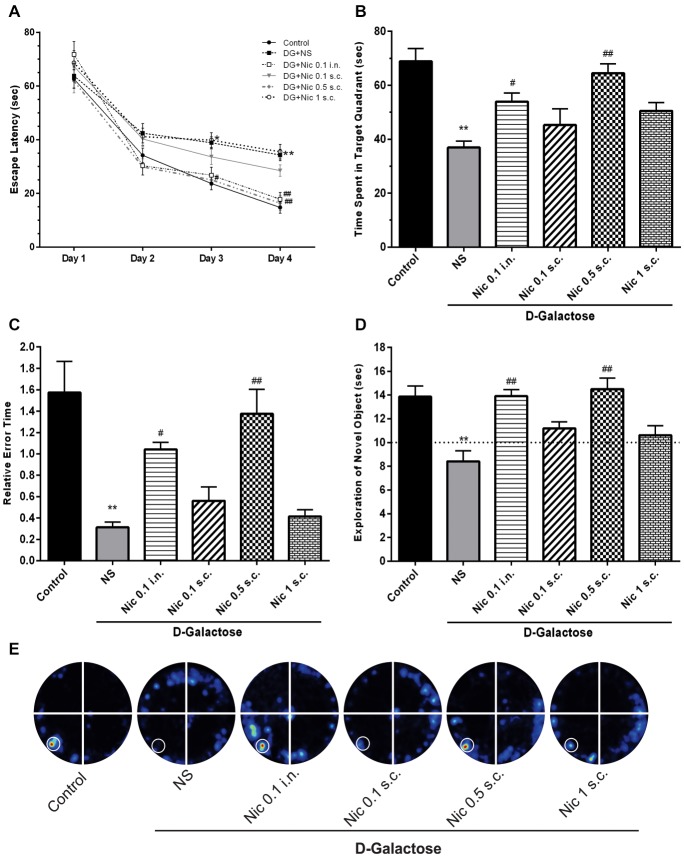
Learning and memory profile in study groups. **(A)** Mean escape latency time during 4 days of training session of MWM, **(B)** mean time spent in the target quadrant in the probe trial session of MWM, **(C)** mean relative error time in the probe trial session of MWM, **(D)** exploration time of the novel object during the retention session of NOR test, **(E)** corresponding heat maps show the combined traces of the mice from each group during the probe test of MWM (left lower part was considered as target quadrant). Values represent the mean ± SEM, (*n* = 12). Significant differences tested by two- **(A)** or one-way ANOVA followed by Tukey’s *post hoc* test; *p < 0.05 and **p < 0.01 compared to the control group. ^#^*p* < 0.05 and ^##^*p* < 0.01 compared to the DG+NS group; NS, normal saline; DG, *D*-galactose; Nic, nicotine; s.c., subcutaneous; i.n., intranasal; MWM, Morris water maze; NOR, novel object recognition.

In the Barnes maze training session, when the mean escape latency was analyzed across the 4 days of training and treated groups, a two-way ANOVA revealed significant effects of group (*F*_(5,264)_ = 12.38, *p* < 0.001), day (*F*_(3,264)_ = 191.7, *p* < 0.001) and group-day interaction (*F*_(15,264)_ = 1.778, *p* = 0.037). Our results showed that chronic DGal+NS administration delayed the finding of escape box in the 3rd and 4th days of the training session in comparison to the control group (*p* < 0.05, and *p* < 0.01, respectively). Conversely, chronic administration of nicotine significantly decreased escape latency time on the 3rd and 4th day of the training session at 0.5 mg/kg s.c. dose (*p* < 0.05 and *p* < 0.01 respectively) and on the 4th day at 0.1 mg/kg i.n. dose compared to the NS-received DGal group (*p* < 0.01; Figure 3A).

Also, one-way ANOVA revealed a significant difference in the time spent in the target quadrant between groups in the probe session (*F*_(5,66)_ = 11.331, *p* < 0.001). *Post hoc* analysis showed that chronic injection of DGal+NS remarkably decreased the time spent in the target quadrant compared to the control group (*p* < 0.01). Also, it was shown that chronic administration of at 0.5 mg/kg s.c. and 0.1 mg/kg i.n. doses significantly increased the time spent in the target quadrant compared to the NS-received DGal group (*p* < 0.01 and *p* < 0.05 respectively; Figures 3B,E).

We found a significant difference between groups in mean relative error time (*F*_(5,66)_ = 13.465, *p* < 0.001). We also found that mean relative error time was significantly lower in the DGal+NS group in comparison to the control group (*p* < 0.01). Chronic nicotine administration increased mean relative error time at 0.5 mg/kg s.c. and 0.1 mg/kg i.n. doses in comparison to the NS-received DGal group (*p* < 0.01 and *p* < 0.05 respectively; Figure 3C).

#### Novel Object Recognition Test

During the training phase, the exploratory preference for the objects was not influenced by the treatments (Data not shown; *p* > 0.05). In the retention phase of NOR test, analysis showed a significant difference between groups in the exploratory preference of the animals (*F*_(5,66)_ = 9.263, *p* < 0.001). Chronic DGal+NS injection significantly decreased the exploratory preference of the animals for the novel object compared to the control group (*p* < 0.01). Additionally, chronic nicotine administration at 0.5 mg/kg s.c. and 0.1 mg/kg i.n. doses remarkably decreased DGal-induced recognition memory impairment compared to the DGal+NS -received group (*p* < 0.01).

All values were substantially different from the chance exploration (10 s) in the retention (*p* < 0.05), but not training phase (*p* > 0.05) as illustrated by the dashed line in the Figure 3D.

### Mitochondrial and Apoptotic Profile

**Figure 4:**
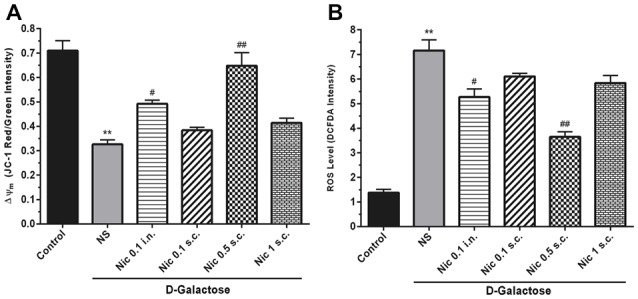
Mito-oxidative profile in study groups. **(A)** Mean of JC-1 red to green intensity as mitochondrial membrane potential (Δψ*m*) index, **(B)** ROS level, Each bar represents the mean ± SEM, (*n* = 8). Significant differences tested by one-way ANOVA followed by Tukey’s *post hoc* test; **p < 0.01 compared to the control group. ^#^*p* < 0.05 and ^##^*p* < 0.01 compared to the DG+NS group; ROS, reactive oxygen species; NS, normal saline; DG, DGal; Nic, nicotine; s.c., subcutaneous; i.n., intranasal.

We found a significant difference between study groups in MMP (*F*_(5,42)_ = 25.164, *p* < 0.001). A significant decrease in the MMP was revealed by *post hoc* analysis in the DGal+NS group in comparison to the control group (*p* < 0.01). Also, treatment with nicotine at 0.5 mg/kg s.c. and 0.1 mg/kg i.n. doses remarkably increased MMP in comparison to the NS-received DGal group (*p* < 0.01 and *p* < 0.05 respectively; Figure 4A).

Also, we found a significant difference between groups in the ROS level, (*F*_(5,42)_ = 54.811, *p* < 0.001). Also, chronic administration of DGal+NS increased ROS level compared to the control group (*p* < 0.01). In addition, chronic nicotine treatment at 0.1 mg/kg i.n. and 0.5 mg/kg s.c. reduced ROS level in comparison to NS-received DGal group (*p* < 0.05 and *p* < 0.01 respectively; Figure 4B).

**Figure 5:**
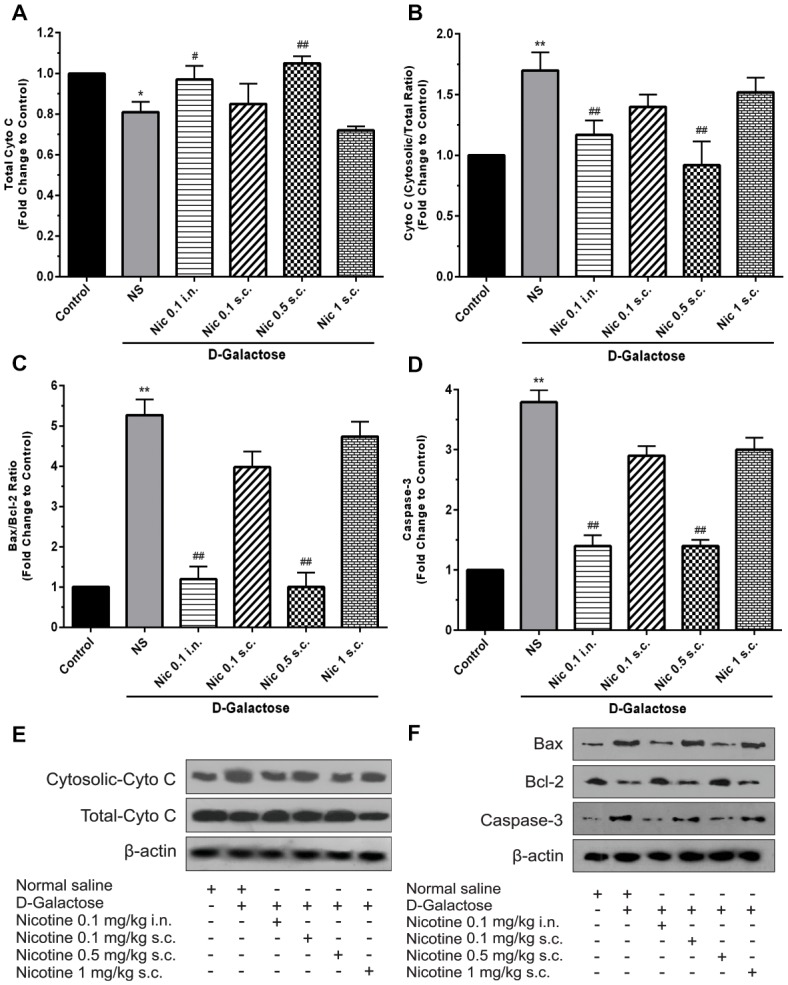
Brain cytochrome C and apoptosis markers levels in different study groups **(A)** mean total cytochrome C, **(B)** cytosolic to total cytochrome C level, **(C)** mean fold change of Bax to Bcl-2 ratio, **(D)** mean fold change of caspase-3 levels, **(E)** representative image of total and cytosolic cytochrome c, **(F)** representative image of Bax to Bcl-2 protein and caspase-3. Each bar represents the mean ± SEM, (*n* = 6). Significant differences tested by one-way ANOVA followed by Tukey’s *post hoc* test; *p < 0.05 and **p < 0.01 compared to the control group; ^#^*p* < 0.05 and ^##^*p* < 0.01 compared to the DG+NS group; NS, normal saline; DG, *D*-galactose; Nic, nicotine; s.c., subcutaneous; i.n., intranasal.

Analysis showed a significant difference between groups in total cytochrome C levels of the brain tissue (*F*_(5,30)_ = 14.87, *p* < 0.001). Chronic administration of DGal+NS decreased total cytochrome C levels compared to the control group in the mice brain (*p* < 0.05). However, chronic administration of nicotine at 0.1 mg/kg i.n. and 0.5 mg/kg s.c. doses increased total cytochrome C level in comparison to the NS-received DGal group (*p* < 0.05 and *p* < 0.01 respectively; Figures 5A,E).

A significant difference was revealed by one-way ANOVA in cytosolic to total cytochrome C ratio (*F*_(5,30)_ = 24.34, *p* < 0.001). Accordingly, *post hoc* analysis showed that chronic DGal+NS administration increases cytosolic to total cytochrome C ratio in comparison to the control group (*p* < 0.01). On the other hand, chronic administration of nicotine at 0.1 mg/kg i.n. and 0.5 mg/kg s.c. doses decreased this item compared to the NS-received DGal group (*p* < 0.01; Figures 5B,E).

We found a significant difference between study groups in Bax/Bcl-2 ratio (*F*_(5,30)_ = 149.7, *p* < 0.001) and caspase-3 levels (*F*_(5,30)_ = 133.2, *p* < 0.001). Subsequently, analysis showed that chronic DGal+NS injection increased Bax/Bcl-2 ratio and caspase-3 levels compared to the control group (*p* < 0.01). In addition, chronic nicotine treatment at 0.1 mg/kg i.n. and 0.5 mg/kg s.c. doses decreased Bax/Bcl-2 ratio and caspase-3 levels compared to the NS-received DGal group (*p* < 0.01; Figures 5C,D,F).

### Neurotrophic Factors

**Figure 6:**
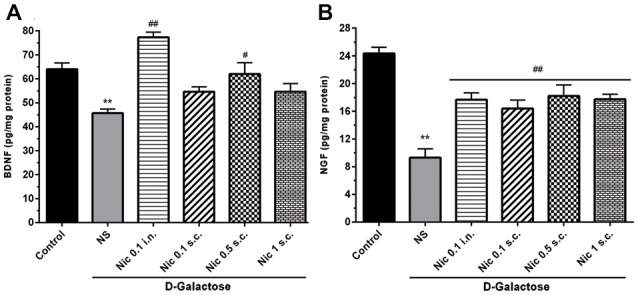
Neurotrophic factors level in study groups. **(A)** BDNF and **(B)** NGF level in different groups. Each bar represents the mean ± SEM, (*n* = 8). Significant differences tested by one-way ANOVA followed by Tukey’s *post hoc* test; **p < 0.01 compared to the control group, ^#^*p* < 0.05 and ^##^*p* < 0.01 compared to the DG+NS group; BDNF, brain-derived neurotrophic factor; NGF, nerve growth factor; NS, normal saline; DG, *D*-galactose; Nic, nicotine; s.c., subcutaneous; i.n., intranasal.

We found a significant difference in the BDNF (*F*_(5,42)_ = 13.24, *p* < 0.001) and NGF (*F*_(5,42)_ = 17.39, *p* < 0.001) levels in the brain tissue in study groups. Chronic treatment with DGal+NS decreased BDNF and NGF levels in comparison to the control group (*p* < 0.01). However, chronic nicotine administration at 0.1 mg/kg i.n. and 0.5 mg/kg s.c. doses increased BDNF level compared to the DGal+NS group (*p* < 0.01 and *p* < 0.05; Figure 6A). Also, chronic nicotine administration at all doses increased NGF level compared to the NS-received DGal group (*p* < 0.01; Figure 6B).

## Discussion

Here, we showed that chronic administration of nicotine at doses of 0.1 mg/kg intranasally and 0.5 mg/kg subcutaneously decreased cognitive impairment induced by chronic administration of DGal to mice. We interpret the results as effects of nicotine against oxidative damage, apoptosis and neurodegenerative lesions in the mouse brain. Nicotine at these doses caused no detectable withdrawal signs in the mice. In contrast, the highest dose of nicotine (1 mg/kg) administered subcutaneously not only had no positive effects on cognition or neurochemical factors in brain tissue but also elicited signs of withdrawal in the treated mice. The lowest dose of nicotine (0.1 mg/kg) administered subcutaneously, in turn, had no effects on cognitive function or signs of withdrawal of the mice.

Age-related memory and learning impairment is thought to be the result of increased neurodegeneration and decline in the neuronal function (Haddadi et al., 2014). The resulting cognitive decline is a multifactorial process and involves oxidative stress, altered brain neurotransmitters (Haider et al., 2014), apoptosis (Kim et al., 2010), mitochondrial dysfunction, et cetera (Martínez et al., 2000).

The DGal model of brain aging is widely-used in rodent studies of features of human brain aging (Haider et al., 2015; Gong et al., 2016; Pourmemar et al., 2017) such as cognitive function impairment (Lu et al., 2010b; Han et al., 2014). The Barnes maze task tested the spatial learning and reference memory associated with hippocampal function in the animals. The Barnes task is less anxiogenic than other tests that assess spatial memory (Harrison et al., 2009) and is widely used to assess cognitive performance in aged animals. Also, the NOR task was used to assess recognition memory that is linked to connections between hippocampus and cortex and is impaired during aging (Gallagher et al., 1993; Sadigh-Eteghad et al., 2015b). These tests have been formerly used to assess rodent cognitive function in neurodegenerative disorders such as AD and their responses to various treatments (O’Leary and Brown, 2009; Sadigh-Eteghad et al., 2015b).

The results showed that chronic administration of DGal with NS raised the escape latency time and decreased the time spent in the target quadrant of the Barnes maze test. The results indicate impairment of working and reference memory in the DGal model of aging in mice, in agreement with results obtained by Ashour et al. (2016). Consistent with other studies (Wei et al., 2005; Pourmemar et al., 2017), we found that long-term administration of DGal with NS shortened explorations of novel object in comparison to the control group, indicating impairment of recognition memory in NS-treated DGal mice.

Chronic nicotine treatment at 0.1 mg/kg i.n. and 0.5 mg/kg s.c. doses reversed the DGal-induced cognitive impairment both in Barnes maze and NOR tests. However, nicotine administration at 0.1 and 1 mg/kg s.c. doses did not have a statistically significant effect on the cognitive performance of the mice in Barnes and NOR tests. Similarly, in a study by French et al. (2006), it was revealed that chronic administration of nicotine to aged rats at 0.1 mg/kg and 0.3 mg/kg doses improved working and reference memories in Morris water maze (MWM) task. This study also showed that cognition-improving effect of nicotine was “dose-dependent or inverted U shape” and certain higher doses had superior effects on the cognitive function (French et al., 2006). Another study showed that effects of nicotine on cognitive function are dependent upon “duration/magnitude of nicotine exposure” and its procognitive impacts are limited under specific conditions (Ortega et al., 2013) which may explain why high (1 mg/kg s.c.) and low (0.1 mg/kg s.c.) doses used in this study did not have cognition-improving effects on mice. Similar studies using the same dose of nicotine, but shorter periods of administration have yielded the same results in the aged rodent both in MWM and NOR tests (Socci et al., 1995; Riekkinen and Riekkinen, 1997; Puma et al., 1999). In a study by Levin and Torry (1996), it was found that acute nicotine administration reduces cognitive deficits in aged rats. However, chronic nicotine injection did not have the same procognitive effects. On the other hand, Arendash et al. (1995) found that chronic nicotine treatment decreases learning/memory deficits in aged rats, and the effects were reproduced in several cognitive tasks. Similarly, Buccafusco and Jackson (1991) showed that nicotine injection in aged monkeys improves their performance in delayed matching-to-sample task.

We could not find any study regarding effects of i.n. nicotine on the cognitive performance of aged animals. In this study, we showed that none of the treatments had effect on the locomotor activity of the mice. These findings were in line with the findings of previous works (Kalejaiye et al., 2013; Pourmemar et al., 2017).

The results also revealed that chronic DGal administration remarkably decreased the number of OAEs in EPM and distance moved in the center in the open filed test suggesting the anxiogenic effects of DGal-induced aging on mice. Of direct relevance to this line of argument is a study by Bessa et al. (2005), demonstrating that aging is accompanied by increased signs of anxiety (Torres et al., 2013). Of the administered doses of nicotine in this study, nicotine at 1 mg/kg s.c. dose significantly increased withdrawal signs including somatic signs and thermal hyperalgesia. It also decreased OAE as well as OAT in EPM and reduced time spent in the arena center in the open field test all of which indicate nicotine potential to increase withdrawal signs and induce dependency at this dose. In line with that, a study by Malin et al. (1992) showed that administration of nicotine at 3 mg/kg dose induced withdrawal signs and thus might cause dependency in mice. In another study, they stated that 1–3 mg/kg daily dose of nicotine could result in dependency in rodents (Malin and Goyarzu, 2009). Other nicotine doses used in this study did not increase withdrawal signs in mice. However, in a study by Grabus et al. (2005), it was shown that chronic oral administration of nicotine (~0.1 mg/kg) increased withdrawal signs presented by the animal on days 1, 2, 3, but not 5. It is believed that “rapid drug delivery” and the concentration of the delivered drug are two main determining factors in the nicotine-induced dependency (Schneider et al., 1996). So, it appears that the administered doses of nicotine in this study (except 1 mg/kg s.c.) due to the specific rate of delivery and concentration of nicotine in the brain did not produce withdrawal signs in mice.

Our data demonstrated that chronically administered DGal increases ROS levels in the mice brain. In line with that many studies have found that chronic DGal administration increases ROS levels and causes oxidative stress in the brain (He et al., 2009; Kumar et al., 2010; Hao et al., 2014) which then predisposes the brain to neurodegeneration and aging (Floyd and Hensley, 2002; Majdi et al., 2016).

We also showed that chronic nicotine administration at 0.1 mg/kg i.n. and 0.5 mg/kg s.c. doses significantly reduced brain ROS levels in mice. In a study by Linert et al. (1999), the authors concluded that nicotine at 0.8 mg/kg i.p. dose does not change ROS levels in the brain. They also stated that the anti-oxidant properties of nicotine might be mediated through its blocking effects on Fenton’s reaction. Also, it has been shown that nicotine may have dual effects on oxidative stress and ROS formation in the brain depending upon the administered dose. It is believed that nicotine has “inverted U-shaped dose-response curves” meaning high doses increase oxidative stress whereas medium doses show antioxidant properties (Guan et al., 2003).

MMP perturbations may have a role in the progression of aging (Reddy and Beal, 2008). Similar to the Shen et al. (2014) results, the findings of the present study showed that chronic DGal administration decreases MMP and thus increases mito-oxidative damage. It is believed that decrease in the MMP increases mitochondrial permeability which subsequently increases ROS and cytochrome C release from mitochondria and results in apoptosis as well as neuronal death (Pollack and Leeuwenburgh, 2001; Zhang et al., 2010). Evidence suggests that ROS formation does not occur until MMP changes which proves MMP importance in the DGal-induced mito-oxidative stress (Nohl et al., 2005). Conversely, some studies have found that increase in the ROS levels causes mitochondrial membrane anisotropy and apoptosis. In fact, increased oxidative stress and ROS levels causes MMP collapse, and transient ROS release to the cytosol which triggers ROS reproduction and is called “ROS-induced ROS-release” (Zorov et al., 2006). We found that chronic nicotine administration at 0.1 mg/kg i.n. and 0.5 mg/kg s.c. doses reduce mito-oxidative damage through decreasing mitochondrial membrane anisotropy. In line with that Cormier et al. (2003), reported that chronically administered nicotine at 0.6 mg/kg dose had protective effects on mitochondria and prevented mitochondrial membrane anisotropy and mito-oxidative damage which happens in neurodegeneration and aging (Cormier et al., 2003; Reddy and Beal, 2008).

In this study, we also showed that chronic injection of DGal increases cytoplasmic to total cytochrome C ratio and decreases total cytochrome C levels in the brain. It also increased Bax/Bcl-2 ratio and caspase-3 levels in DGal-received mice brain. As we discussed earlier DGal treatment increases ROS levels in the brain which leads to MMP perturbations. This subsequently increases cytochrome C release to the cytoplasm. Cytoplasmic cytochrome C then links to other apoptotic factors and creates an apoptosome which then activates caspase-3 (Pollack and Leeuwenburgh, 2001; Pollack et al., 2002). In addition, DGal-induced increase in the Bax/Bcl-2 ratio further increase cytochrome C release to the cytoplasm and exacerbates the above-mentioned pathologic pathway. These mechanisms finally result in neuronal apoptosis as an important component of brain aging and neurodegeneration (Beal, 2005). DGal administration also decreased total cytochrome C levels which may indicate an age-related decrease in the number of active mitochondria (Navarro et al., 2002; O’Toole et al., 2010).

In this study, administration of nicotine at 0.5 mg/kg s.c. and 0.1 mg/kg i.n. doses was found to reverse the mentioned pathologic events. Nicotine decreased Bax/Bcl-2 ratio as well as cytochrome C release to the cytoplasm. This along with decreased caspase-3 levels is thought to prevent the subsequent apoptosis in the DGal-received mice brain. In line with that, nicotine has been shown to be the so-called “survival agonist” and inhibit pro-apoptotic pathways (Mai et al., 2003; Tizabi et al., 2005). In a direct relevance to this line of evidence, Marrero and Bencherif (2009) showed that nicotine increases the production of Bcl-2 and blocks the release of cytochrome C the cytosol and prevents apoptosis. Nicotine at the mentioned doses also increased total cytochrome C levels suggesting nicotine-induced improvement of mitochondrial function.

We confirmed that long-term DGal injection reduces NGF and BDNF levels as neuroprotective factors in the brain. Woo et al. (2014) found that chronic DGal administration impairs neurotrophic factors production in the brain resulting in cognitive impairment. Similarly, Erraji-Benchekroun et al. (2005) showed that the production of neurotrophic factors declines through time in the aging brain. The results of this study also showed that nicotine treatment chronically at 0.5 mg/kg s.c. and 0.1 mg/kg i.n. doses can increase BDNF levels in the DGal-received mice brain. Also, all the administered doses of nicotine increased NGF level in this aging model.

Nicotine-mediated enhancement of nuclear translocation and transcriptional activity of NF-κB amplifies the expression of NGF and protects the brain from aging-induced neuronal damage (Wongtrakool et al., 2014). Additionally, nicotine regulates NGF level possibly via glutamatergic neurones modulation (Rattray, 2001). The increased NGF levels in the brain could then improve learning, memory and cognitive performance (Fischer et al., 1991; De Rosa et al., 2005) which is impaired in aging. Also, evidence suggests that chronic nicotine administration at 0.5 mg/kg i.p. dose (Kenny et al., 2000) increases expression of BDNF through *α*_7_ nAChRs-mediated pathways. BDNF then involves in memory formation in the hippocampus and long-term potentiation (Tyler et al., 2002; Czubak et al., 2009).

Intranasal drug delivery is a novel and safe way to administer medications through the nasal mucosa (Farzampour et al., 2016). This route of drug administration shunts the BBB, is non-invasive and encompasses two separate pathways including trans-neuronal or immediate and para-neuronal or delayed (Mustafa et al., 2016). It has been shown that nicotine nasal spray improves continuous attention, working memory, and executing processing (Smith et al., 2002; Myers et al., 2008). Our results suggested the anti-aging effect of intranasal nicotine on mice through its anti-apoptosis, neuroprotective and anti mito-oxidative actions without increasing withdrawal signs presentation and causing dependency. This might be an alternative approach in the treatment of age-associated cognitive impairment.

In conclusion, this set of data showed that nicotine at certain controlled doses has a potential to attenuate age-induced cognitive impairment without producing withdrawal signs and dependency. These doses could also ameliorate age-induced mito-oxidative damage, apoptosis and neurotrophic factors level reduction. Further, our results indicated that i.n. nicotine delivery could be an alternative choice for the treatment of age-related cognitive decline.

## Author Contributions

AM, FF, ME and JM performed the experiments, interpreted the results and wrote the manuscript. AG, SS-E and MT designed the experiments. AG critically interpreted data and critically revised and approved the manuscript.

## Conflict of Interest Statement

The authors declare that the research was conducted in the absence of any commercial or financial relationships that could be construed as a potential conflict of interest.
